# Long-term cattle manure addition enhances soil-available phosphorus fractions in subtropical open-field rotated vegetable systems

**DOI:** 10.3389/fpls.2023.1138207

**Published:** 2023-03-13

**Authors:** Yanting Mao, Wei Hu, Yongmei Li, Yuan Li, Baokun Lei, Yi Zheng

**Affiliations:** ^1^ Faculty of Plant Protection, Yunnan Agricultural University, Kunming, China; ^2^ Institute of Agricultural Environment and Resources, Yunnan Academy of Agricultural Sciences (YAAS), Kunming, China; ^3^ The New Zealand Institute for Plant and Food Research Limited, Canterbury Agriculture and Science Centre, Christchurch, New Zealand; ^4^ Faculty of Resource and Environment, Yunnan Agricultural University, Kunming, China; ^5^ National Field Scientific Observation and Research Station of Grassland Agro-Ecosystems in Gansu Qingyang, College of Pastoral Agriculture Science and Technology, The State Key Laboratory of Herbage Improvement and Grassland Agro-ecosystems of Lanzhou University, Lanzhou, China; ^6^ Department of President Office, Yunnan Open University, Kunming, China

**Keywords:** phosphorus fractions, phosphate use efficiency, manure, vegetable yield, subtropical

## Abstract

**Introduction:**

Evaluation of the changes in phosphorus (P) fractions (various P forms) and their availability at different soil layers is critical for enhancing P resource use efficiency, mitigating subsequent environmental pollution, and establishing a suitable manure application strategy. However, changes in P fractions at different soil layers in response to cattle manure (M), as well as a combined cattle manure and chemical fertilizer application (M+F), remain unclear in open-field vegetable systems. If the amount of annual P input remains the same, identifying which treatment would cause a higher phosphate fertilizer use efficiency (PUE) and vegetable yield while simultaneously reducing the P surplus is especially warranted.

**Methods:**

Based on a long-term manure experiment that started in 2008, we used a modified P fractionation scheme to analyze P fractions at two soil layers for three treatments (M, M+F, and control without fertilizer application) in an open-field cabbage (Brassica oleracea) and lettuce (Lactuca sativa) system, and assessed the PUE and accumulated P surplus.

**Results:**

The concentrations of the soil P fractions were higher in the 0–20-cm soil layer compared to the 20–40-cm layer, except for organic P (Po) and residual-P. M application significantly increased the inorganic P (Pi) (by 8.92%–72.26%) and the Po content (by 5.01%–61.23%) at the two soil layers. Compared with the control and M+F treatments, M significantly increased residual-P, Resin-P, and NaHCO3-Pi at both soil layers (by 31.9%–32.95%, 68.40%–72.60%, and 48.22%–61.04%), whereas NaOH-Pi and HCl-Pi at 0–20 cm were positively correlated with available P. Soil moderately labile-P was the predominant P component in the two soil layers (accounting for 59%–70%). With the same annual P input amount, M+CF created the highest vegetable yield (117.86 t ha-1), and PUE (37.88%) and M created the highest accumulated P surplus (128.80 kg ha^-1^yr^-1^).

**Discussion:**

Collectively, a combined manure-chemical fertilizer application has great potential to yield a long-term positive outcome both in terms of vegetable productivity and environmental health in open-field vegetable systems. This highlights the methods’ benefits as a sustainable practice in subtropical vegetable systems. Specific attention should be given to a P balance to avoid excessive P input if a rational strategy for manure application is to be attained. This is especially the case for stem vegetables that require manure application and decreases the environmental risk of P loss in vegetable systems.

## 1 Introduction

Phosphorus (P) as a crucial macro-element maintains healthy crop growth and enters the soil through P minerals (Filippelli, 2008; Augusto et al., 2017). A strong soil P adsorption capacity impedes P use efficiency (George et al., 2017). To meet the demands of plant growth while simultaneously enhancing yield, excessive P fertilizer was traditionally applied to agricultural fields, particularly when cultivating plants with short growth phases and high yields (Yan et al., 2013). However, a P surplus leads to soil P accumulation, which can lead to eutrophication of the local environment through leaching and runoff into water bodies (Kang et al., 2018; Zhang et al., 2018). In recent years, many major rivers across the world (i.e., the Danube River in Europe, the Ganges in India, Indus River in Pakistan, and the Yellow River and Yangtze in China) have suffered from P pollution due to inadequate agricultural activities (Mekonnen and Hoekstra, 2018). As a consequence, the impacts of agricultural P application regimes have attracted increasing attention.

In general, P is characterized by various complex forms and different functional roles. To a large extent, P availability and conversion cycles are determined by various soil P fractions (Helfenstein et al., 2018a; Helfenstein et al., 2018b). Specifically, inorganic P (P_i_) is a dominant form (40%–90%), and only its soluble part can directly be assimilated by plants. Organic P (P_o_), accounts for 10%–60% of the total amount of phosphorus (TP) in the soil (Hayes et al., 2000) and can only be assimilated by plants after having been mineralized by microorganisms (Frossard et al., 2000; Chiara et al., 2018). The various soil P fractions differ in how they are assimilated by plant roots. Additionally, soil P can be classified into labile, moderately labile, and non-labile P according to its availability to plants (Redel et al., 2019). The most available soil P fractions (Resin-P, NaHCO_3_-P_i_, and NaHCO_3_-P_o_) are also the most transformed P fractions following P fertilizer application (Tiessen and Moir, 1993). The P_o_ pool is crucial for the long-term supply of available P without fertilizer addition, e.g., moderately labile-P (NaOH-P_o_) and low-labile-P (concentrated HCl-P_o_; abbreviated as “Conc.HCl-P_o_”; Randhawa et al., 2005). In subtropical regions, the applied P fertilizer is rapidly transformed into unavailable P by soil colloids (Zhang et al., 2021). In calcareous soils, P is usually absorbed by Ca^2+^ and Mg^2+^, whereas it can easily be fixed by Al^3+^ and Fe^2+^ in acidic soils (Hedley et al., 1982).

Previous studies on changes in P fractions have reported inconsistent results and mainly focused on the effect of nitrogen (N) addition on soil P availability in staple croplands or changes in the compositional proportions of P in different rotational systems (Mahmood et al., 2021). For example, the transformed proportion of residual-P was highest after a 17-year P fertilizer application in a wheat (*Triticum aestivum*) – maize (*Zea mays*) rotational system (Mao et al., 2015). In contrast, an intensive vegetable system study suggested that the proportion of labile-P (Resin-P, NaHCO_3_-P_i_, and NaHCO_3_-P_o_) increased with 10–15 years of P fertilizer application (Zhang et al., 2021). Moreover, changes in P fractions were also found to be related to the number of years of fertilizer application and varied with soil depth (Zhang et al., 2021). However, the changes that occur in P fractions at different soil layers under long-term fertilizer application in open-field intensive vegetable production systems are still unclear, particularly with the application of cattle manure (M). The transformation of soil P can largely be influenced by organic amendments through complex soil physiological, chemical, and biological mechanisms (Steiner et al., 2007; Malik et al., 2012; Lin et al., 2019) to secure and increase soil fertility, crop yield, and food quality (Bouwman et al., 2012; Sun et al., 2015).

In intensive vegetable production systems, the P inputs (117 kg P ha^-1^) per season are generally higher than the P outputs (25 kg P ha^-1^) (Yan et al., 2013). Recently, P fertilizer addition was banned in the Erhai Lake watershed area to protect its water quality since open-field vegetable systems are widespread in this region. As a result, manure, which has a high organic matter (OM) concentration and P availability (Penuelas et al., 2013), has been promoted as an alternative to synthetic fertilizer. However, organic amendments are usually applied based on N agronomic rates, which results in considerable soil residual P accumulation (Sharpley et al., 2007). Manure application also increases P saturation in soil sorption sites (Song et al., 2007). Excessive manure application can cause water eutrophication when P migrates from the topsoil to the groundwater or surface waters (Yuan et al., 2018; Qin et al., 2020). Optimal manure management can activate soil P availability and reduce the need to apply synthetic P fertilizer (Bi et al., 2020). An inflexion point model of the relationship between CaCl_2_-P (soluble soil P) and Olsen-P (available soil P) is often used to predict the P release potential of soils (Khan et al., 2018). Thus, to enhance P resource use efficiency and mitigate subsequent environmental pollution, an equilibrated P budget adjustment for phosphate fertilizer use efficiency (PUE) is necessary for establishing a suitable manure application strategy. The long-term impacts of manure application on different soil layer P fractions, as well as the redistribution of surplus P fertilizer, should therefore be determined in vegetable production systems. This is especially critical in subtropical highland monsoon-type climate watersheds, where vegetables are planted widely and where the potential for P loss is high.

In this context, the present study had the following aims: (1) to evaluate the changes in nine specific P fractions for different soil layers driven by a 14-year continuous M application regime, (2) to reveal the relationships among various soil, vegetable, and P fractions in response to M application in the vegetable soils of the Erhai watershed, (3) to determine whether soil P fractions and soil-available P concentrations are correlated, and (4) to compare the PUE of manure, or a manure-chemical fertilizer (F) combination, to determine an optimized yield and a minimized P surplus.

## 2 Materials and methods

### 2.1 Study site

The study site was located in Dali, Yunnan, China (25°9′45´N, 100°12′14.3´E), and has been managed by the Yunnan Academy of Agricultural Sciences since 2008. This region is a subtropical highland with a monsoon-type climate, with an average annual temperature of 16.5°C and an average annual precipitation of 1,100 mm. The soil is classified as loamy soil, with a pH of 5.6, OM of 38.4 g kg^-1^, bulk density of 0.98 g cm^-3^, total N of 2.3 g kg^-1^, total potassium (K) of 20.1 g kg^-1^, total P of 1.5 g kg^-1^, and Olsen-P of 52.66 mg kg^-1^. These parameters were determined at the start of the experiment in 2008.

The experimental site consisted of a cabbage-lettuce (*Brassica oleracea* var. *capitata*-*Lactuca sativa* var. *asparagina*, *augustan*) rotational system, with different fertilizers having been applied since 2008. Cabbage plants were transplanted at a depth of 5 cm in early May, whereas lettuce plants were transplanted in late August, at about 60,450 plants ha^-1^ for each cultivation period. Every year, cabbage was harvested in the middle of July, and lettuce was harvested in the middle of October. Both vegetables were managed in accordance with local farming practices.

### 2.2 Study design

Based on a preliminary experiment, three treatments were chosen and structured into a randomized complete block design, with each combination done in triplicate. Each plot was 24 m^2^ (4 × 6 m), and the three treatments were as follows: (1) CK: control without fertilizer (N: P: K_2_O = 0); (2) M: single cattle manure application (cabbage/lettuce per season N: 665 kg ha^-1^, P: 100 kg ha^-1^, K_2_O: 702 kg ha^-1^); and (3) M+CF: cattle manure-chemical fertilizer combination application, in which manure (27% P) was combined with chemical P fertilizer (73% P), with the addition of normal fertilization rates (cabbage/lettuce per season N: 554 kg ha^-1^, P: 100 kg ha^-1^, K_2_O: 564 kg ha^-1^). Based on the results of a local survey, M+CF was the optimal fertilizer application amount, and the fertilizer application amount of M was in accordance with the equivalent amount of P for M+CF. Among the three treatments, M and M+CF had the same P application amount. The N (46% urea) and K (50% potassium sulfate) fertilizers were applied as basal and supplementary fertilizers, whereas P (16% calcium magnesium phosphate) and manure were only used as basal fertilizers. The manure was mainly sourced from composted cattle dung (N: 1.99%, P: 0.29%, K_2_O: 2.1%, water content: 55.6%). The nutrient concentrations for M represent the average values over numerous years (see **Table 1** for more details regarding the soil indicators for the three treatments and two soil layers at the end of the study).

**Table 1:** Soil indicators of three treatments in different soil layers.

	0-20 cm			20-40 cm		
Soil indicator Treatment	pH	OM (g kg^-1^)	Olsen-P (mg kg^-1^)	pH	OM (g kg^-1^)	Olsen-P (mg kg^-1^)
CK	7.4 ± 0.2a	24.68 ± 1.5b	64.69 ± 3.3b	6.9 ± 0.3a	20.24 ± 3.6b	61.00 ± 9.2b
M	7.1 ± 0.1a	59.92 ± 2.9a	133.45 ± 5.5a	7.2 ± 0.2a	35.14 ± 5.0a	100.40 ± 5.0a
M+CF	4.3 ± 0.1b	42.37 ± 1.6a	123.52 ± 3.3a	4.1 ± 0.3b	28.85 ± 4.9a	92.81 ± 6.3a

Data of soil indicators presented are of those at the end of the study. Data are means and standard errors (n=3), the different letters in the same column mean significant differences at P< 0.05, respectively. CK is the control treatment.

### 2.3 Sampling and measurements

The current study was based on a 14-year open-field vegetable system. The following metrics were analyzed from 2020 to 2022: vegetable yield, nutrient concentration, soil physicochemical properties, and soil P component fractionation. Fresh vegetable samples from the three treatments were randomly collected during the harvesting season by sampling 10 plants from each plot, which were also used to measure dry weight and total P concentration. Soil samples were randomly collected from five cores in each plot at the same depth and then mixed to form one composite sample; soil samples from two depths (0–20 and 20–40 cm) were collected during the growing and harvesting stages. The two depths were selected based on differences in soil tillage disturbance and root distribution. The 0–20-cm soil depth characterizes the typical plough layer in this area, which is artificially disturbed twice during each vegetable season, and results from tillage and fertilizing application. The root distribution of Chinese cabbage and lettuce is mostly less than 40 cm; thus, roots in the 20–40-layer cm affect nutrient transformation and absorption. All soil samples were air-dried and then passed through a 2-mm sieve, whereafter they were used to determine the concentrations of the soil P fractions, Olsen-P, and CaCl_2_-P.

Soil pH was determined from free deionized water and a soil:water ratio of 1: 2.5, using a pH electrometer. The soil CaCl_2_-P concentration was determined using CaCl_2_ (0.01 mol L^-1^) and a 1:5 soil: solution ratio (Bao, 2000). Soil organic carbon (OC) was determined using the potassium dichromate-sulfuric acid (K_2_Cr_2_O_7_-H_2_SO_4_) oxidation method. The measured value was converted to OM using the following equation: organic matter (OM) (g kg^-1^) = organic carbon (OC) (g kg^-1^) ×1.724 (Bremner and Jenkinson, 1960). Available P (Olsen-P) was extracted with 0.5 mol L^-1^ NaHCO_3_ (Olsen, 1954).

**Figure 1:**
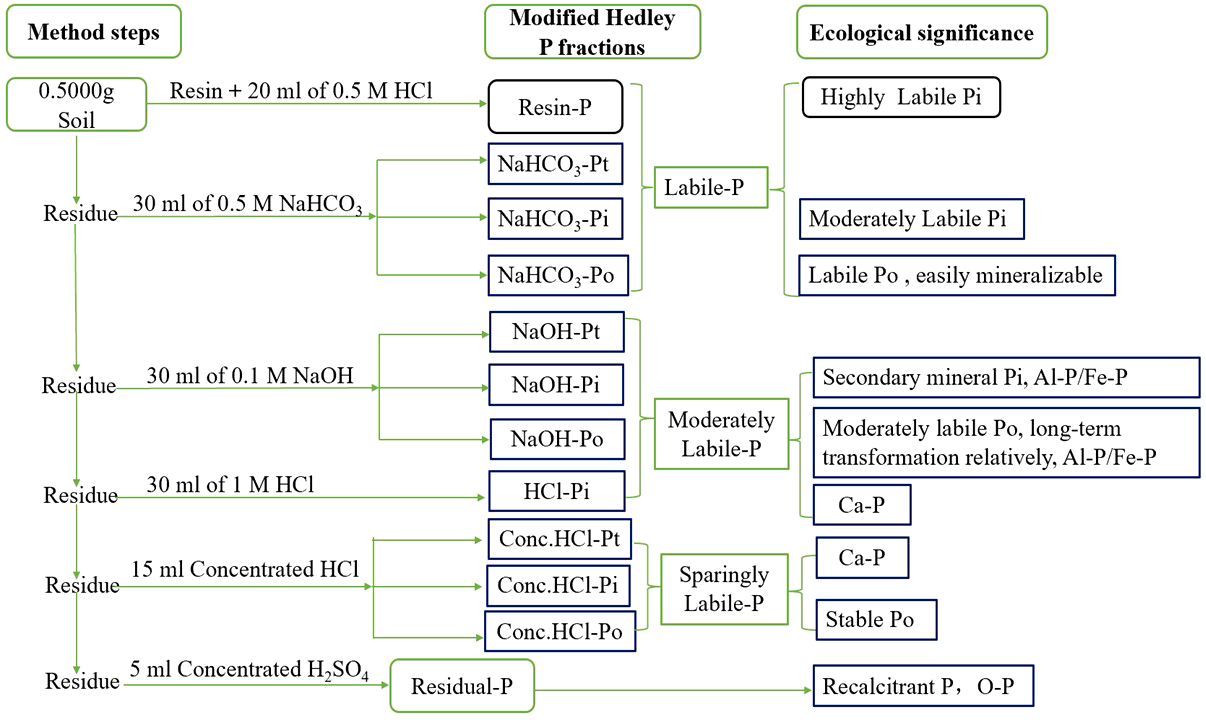
Modified P fractionation scheme, P_i_, P_o_, and P_t_ represent the inorganic, organic and total P, respectively. NaHCO_3_-P_t_=NaHCO_3_-P_i_+NaHCO_3_-P_o_; NaOH-P_t_=NaOH-P_i_+ NaOH-P_o_.

Soil phosphate fractions were measured using a modified fractionation scheme (Hedley et al., 1982; Tiessen and Moir, 1993; see **Figure 1**). The total P (P_t_) was the sum of organic P (P_i_) and inorganic P (P_o_) fractions. The differences in the concentrations of P_t_ and P_i_ were used to calculate the organic P (P_o_) component. For the determination of total P (P_t_) and inorganic P (P_i_), two sets of subsamples were divided from P filtrates extracted with Resin-P, NaHCO_3_-, NaOH-, and concentrated HCl (Conc.HCl-) extractable P, respectively. All P analyses (P_i_ or P_t_) were performed using a UV spectrophotometer.

Briefly, 0.5000 g of soil was extracted with Resin and 20 mL 0.5 M HCl, and 30 mL 0.5 M NaHCO_3_ (pH 8.5), 0.1 M NaOH, and 1 M dilution HCl for 16 h each to extract Resin-P, NaHCO_3_-P, NaOH-P, and HCl-P extractable P fractions, respectively. Subsequently, the remaining soil was extracted with 15 mL of hot (80°C) concentrated HCl (Conc.HCl-P) for 20 min. The final extraction was done by using 5 mL H_2_SO_4_ and H_2_O_2_ at 360°C for residual-P determination.

For the determination of NaHCO_3_-P_i_ and NaOH-P_i_, the filtrate obtained by passing through a 0.45-μm membrane was centrifuged at 8,000 rpm for 8 min at 0°C before analysis. The HCl-P_i_ was directly measured without any pretreatments. For measuring P_t_, another set of subsamples was extracted with ammonium persulfate and 1.8 M H_2_SO_4_ (for NaHCO_3_-P_t_, NaOH-P_t_ and Conc.HCl-P_t_), covered with sterilize gauze, and autoclaved for 60 min (for NaHCO_3_-P_t_ and NaOH-P_t_) or 90 min (for Conc.HCl-P_t_) (Mirabello et al., 2013).

Specifically, the four groups are labile-P (Resin-P, NaHCO_3_-P_i_, and NaHCO_3_-P_o_), moderately labile-P (NaOH-P_i_, NaOH-P_o_, and HCl-P_i_), low-labile-P (Conc.HCl-P_i_ and Conc.HCl-P_o_), and residual-P. Labile-P is a type of plant available-P and occurs mostly on the surfaces of ribonucleic acid and crystalline compounds. Moderately labile-P is a mineral P and contains surface-bound Fe, Al, and Ca cations. The Al-P/Fe-P compound is extracted by NaOH (NaOH-P_i_, NaOH-P_o_). The Ca-P bond compound is extracted by diluted HCl (HCl-P_i_). Low-labile-P is closely associated with Ca phosphates. Residual-P is a recalcitrant, occluded P (O-P) that includes highly unavailable P_i_ and non-labile P_o_ (Tiessen and Moir, 1993; Zhang et al., 2020).

### 2.4 PUE and relevant parameters and calculations

The input P (P_input_) included chemical P fertilizer (P_CheF_; kg P ha^-1^) and manure P (P_Manure_; kg P ha^-1^, Eq. 1). The output P (P_output_) was calculated as the sum of absorption P (kg P ha^-1^) in the harvested vegetables (P_Vyield_) and vegetable waste (P_Vwaste_, Eq. 2). The absorption of P_Vyield_ and P_Vwaste_ was calculated by multiplying vegetable yield (t ha^-1^) by its P concentration (g kg^-1^) and vegetable waste yield (t ha^-1^) by its P concentration (g kg^-1^), respectively. The difference between P input (P_input_) and output (P_output_) was defined as P surplus (P_surplus_, Eq. 3). The phosphate fertilizer use efficiency (PUE, Eq. 4) was calculated as the ratio of P_output_ to P_input_. Accumulated P surplus was calculated by multiplying P surplus each year (P_surplus_, Eq. 3) by fertilizer application years, i.e.,14 years. Accumulated P input and P output were calculated by multiplying the P input each year (P_input_, Eq. 1) by the fertilizer application year and the P output each year (P_output_, Eq. 2) by the fertilizer application year, respectively. The equations are as follows:


(1) $${\text{P}}_{\text{input}}=\;{\text{P}}_{\text{CheF}}+\;{\text{P}}_{\text{Manure}}$$

(2) $${\text{P}}_{\text{output}}={\text{P}}_{\text{Vyield}}+\;{\text{P}}_{\text{Vwaste}}$$

(3) $${\text{P}}_{\text{surplus}}=\;{\text{P}}_{\text{input}}-\;{\text{P}}_{\text{output}}$$

(4) $$\text{PUE }\left(\%\right)\;=\;{\text{P}}_{\text{output}}/\;{\text{P}}_{\text{input}}\times 100\%$$

### 2.5 Data analysis

We performed ANOVAs with Tukey tests at *P* = 0.05 to assess significant differences among treatments (CK, M, M+CF) for the P fractions at the two soil depths (0–20 and 20–40 cm) after harvesting. Redundancy analysis (RDA) was performed to determine the relationships among P fractions, soil, and vegetable properties. All statistical analyses were performed with SPSS 17.0 and Canoco 5.0.

## 3 Results

### 3.1 Long term manure application affected PUE in the open-field rotational vegetable system

The mean annual PUE values and the relevant parameters in the open-field rotational vegetable system were significantly affected by the 14 years of manure application (**Table 2**). Similar results were obtained for the M+CF and M treatments for annual P fertilizer input, P output, P surplus, and PUE. Likewise, similar results were obtained for P output for M+CF and PUE. However, P surplus showed the opposite pattern. In contrast, P output and P surplus were not significantly different between M and M+CF; however, after 14 years of manure application, the accumulated P surplus was significantly higher for M compared to M+CF.

**Table 2:** Mean annual P fertilizer input (including organic P and chemical P input), P removal, and P surplus as affected by 14-year treatments that open-field rotated vegetables had been in production.

Parameter	Unit	CK	M	M+CF
P fertilizer input	kg ha^-1^yr^-1^	0	200	200
Organic P input	kg ha^-1^yr^-1^	0	200	54
Chemical P input	kg ha^-1^yr^-1^	0	0	146
P output	kg ha^-1^yr^-1^	49.68 ± 4.1b	71.20 ± 2.7a	75.75 ± 8.7a
P surplus	kg ha^-1^yr^-1^	-49.68 ± 13.8b	128.80 ± 15.2a	124.25 ± 12.3a
PUE	%	–	35.60 ± 2.7a	37.88 ± 8.7a
Accumulated P input	kg ha^-1^	0	2800	2800
Accumulated P surplus	kg ha^-1^	-695.52 ± 25.6c	1803.20 ± 38.9a	1739.50 ± 12.6b

P output, P surplus are expressed on mean annual basis. These values were calculated on each year over the 14-year experiment (2 crop seasons), data are means standard errors (n=14 years). Accumulated P based on mean of 14 years (n=3). The different letters in the same row mean significant differences at P<0.05, respectively. CK is a control treatment.

The symbol "-" represents that the P fertilizer input as denominator cannot be zero, which is meaningless.

### 3.2 Long-term manure application affected TP, inorganic P (TPi), organic P (TP_o_), Residual-P, and Olsen-P

**Figure 2:**
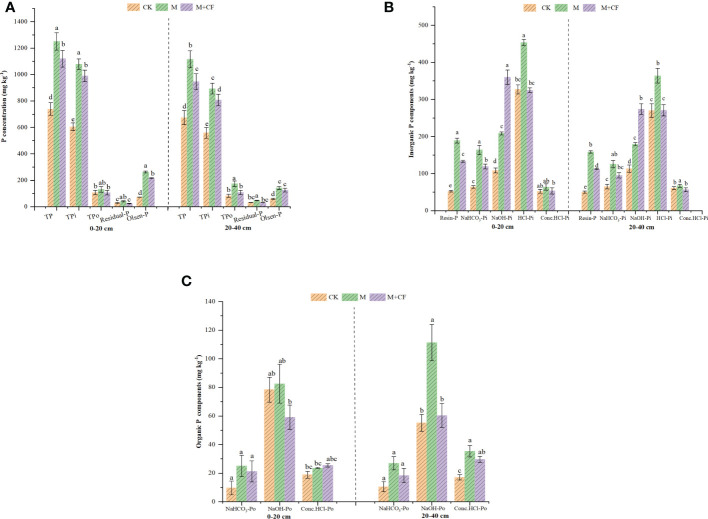
**(A)** Changes in total P (TP), total inorganic P (TP_i_), total organic P (TP_o_), residual-P, and Olsen-P to different fertilizer applications at two soil layers. TP included all TP_i_, TP_o_, and residual-P, and Olsen-P was available P. Bars over the marker show standard error (n=3). Above the columns, lowercase letters indicate significant differences at *P<0.05* of the same indicators (3 treatments) at two soil layers; **(B)** Changes in inorganic P (P_i_) fractions in response to different fertilizer applications at two soil layers. Bars over the marker show standard error (n=3). Above the columns, lowercase letters indicate significant differences at *P<0.05* of same indicators (3 treatments) at two soil layers. Soil P_i_ fractions: labile-P (Resin-P, NaHCO_3_-P_i_); Moderately Labile-P (NaOH-P_i_, HCl-P_i_) and Sparingly Labile-P (Conc.HCl-P_i_); **(C)** Changes in organic P fractions in response to different fertilizer applications at two soil layers. Bars over the marker show standard error (n=3). Above the columns, lowercase letters indicate significant differences at *P<0.05* of the same indicators (3 treatments) at two soil layers. Soil organic P fractions: labile-P (NaHCO_3_-P_o_); Moderately Labile-P (NaOH-P_o_); Sparingly Labile-P (Conc.HCl-P_o_).

Long-term M and M+CF treatments significantly affected TP, inorganic P (TP_i_), organic P (TP_o_), residual-P, and Olsen-P at both soil layers (**Figure 2A**). Regardless of the P fractions, the M treatment always had the highest P concentration (*P< 0.05*), followed by M+CF at both soil layers. In contrast, except for the TP_o_ concentration under the M+CF treatment at the 0–20- (106 mg kg^-1^) and 20–40-cm (108 mg kg^-1^) soil layers, the residual-P concentrations under M+CF (24, 30 mg kg^-1^) at the two soil layers were slightly lower than those in CK, which had the lowest concentration (*P< 0.05*).

### 3.3 Effects of long-term manure application on inorganic P fractions

All inorganic P (P_i_) fractions were significantly different (**Figure 2B**). The highest NaOH-P_i_ concentrations occurred under M+CF (360, 274 mg kg^-1^) at both soil layers (*P< 0.05*). Manure application significantly increased the concentrations of the P_i_ fractions, including Resin-P, NaHCO_3_-P_i_, HCl-P_i_, and Conc.HCl-P_i_ at both soil layers. The mean values of these P_i_ fractions at the 0–20-cm soil layer ranged from 52–453 mg kg^-1^, whereas for the 20–40-cm layer, it ranged from 50–364 mg kg^-1^. Regardless of soil depth, HCl-P_i_ was the dominant P_i_ component under the M and CK treatments, whereas NaOH-P_i_ was the dominant P_i_ component under the M+CF treatment (*P< 0.05*). The Conc.HCl-P_i_ concentration was the lowest, irrespective of fertilizer treatment and soil depth (*P< 0.05*), except for the Resin-P concentration under CK (*P< 0.05*).

### 3.4 Effects of long-term manure application on organic P fractions

The organic P (P_o_) fractions (NaHCO_3_-P_o_ and Conc.HCl-P_o_) were significantly affected by manure application across both soil layers, except for the NaOH-P_o_ concentration (**Figure 2C**). In general, the M treatment increased the concentrations of the P_o_ fractions more than the other two treatments, regardless of soil depth (*P< 0.05*). Particularly, compared with CK, the M treatment significantly increased the NaOH-P_o_ and Conc.HCl-P_o_ concentrations by 50% and 52%, respectively, at the 20–40-cm soil layer. The concentrations of the P_o_ fractions at the 20–40-cm soil layer were higher compared to those at the 0–20-cm layer (*P< 0.05*), and the mean values of the P_o_ fractions at the 0–20-cm and 20–40-cm soil layers ranged from 10–83 mg kg^-1^ and 11–111 mg kg^-1^, respectively. The mean concentrations of NaHCO_3_-P_o_ were the lowest (10–27 mg kg^-1^), irrespective of manure treatment and soil depth (*P< 0.05*).

### 3.5 P activity classification proportion under different manure treatments

**Figure 3:**
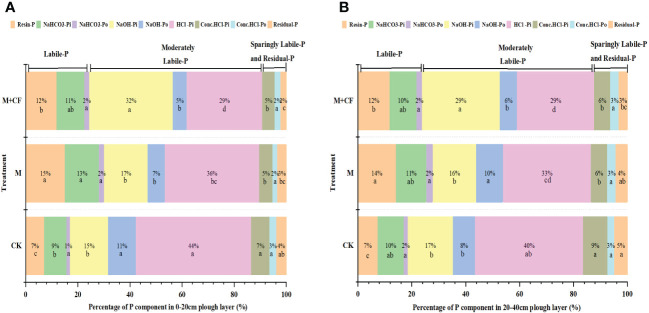
The proportion of P activity classification in response to different fertilizer applications at two soil layers. Labile-P: Resin-P, NaHCO_3_-P_i_, NaHCO_3_-P_o_; Moderately Labile-P: NaOH-P_i_, NaOH-P_o_, HCl-P_i_, Sparingly Labile-P: Conc.HCl-P_i_, Conc.HCl-P_o_ and Residual-P. **(A)** represent 0-20cm soil layers, and **(B)** represent 20-40cm soil layers. Below the proportion, lowercase letters indicate significant differences at *P<0.05* of the same indicators (3 treatments)at two soil layers.

Four P activity levels were originally defined based on P activity (**Figure 1**); specifically, labile-P and moderately labile-P dominated under the three treatments across both soil layers (*P< 0.05*). The proportions of labile-P and moderately labile-P were higher at the 0–20-cm soil layer (*P<0.05*); in contrast, low-labile-P and residual-P were higher at the 20–40-cm layer (*P< 0.05*), whereas the concentrations of labile-P and residual-P were not significantly different (**Figure 3**). Manure treatment (M and M+CF) significantly affected soil labile-P and moderately labile-P, which accounted for 17%/19% and 70%/65% of the total P fractions under the control treatment, 30%/27% and 60%/59% under the M treatment, and 25%/24% and 66%/64% under the M+CF treatment at 0–20 cm and 20–40 cm, respectively (**Figure 3**). In contrast, manure treatment did not significantly affect the corresponding low-labile-P and residual-P concentrations.

Manure application (M and M+CF) significantly increased the proportion of soil labile-P, which was the most available P component for the vegetables, ranging from 8%–13% at 0–20 cm and 5%–8% at 20–40 cm. In contrast, manure application decreased the proportion of moderately labile-P, which ranged from 4%–10% at 0–20 cm and 1%–6% at 20–40 cm (*P< 0.05*). Overall, regardless of treatment and soil depth, inorganic P dominated, accounting for more than 80% of the total extracted P, whereas organic P accounted for less than 15% and residual-P for 5% of the total extracted P (**Figure 3**).

### 3.6 Relationships among soils, vegetable factors, and P fractions with manure application

**Figure 4:**
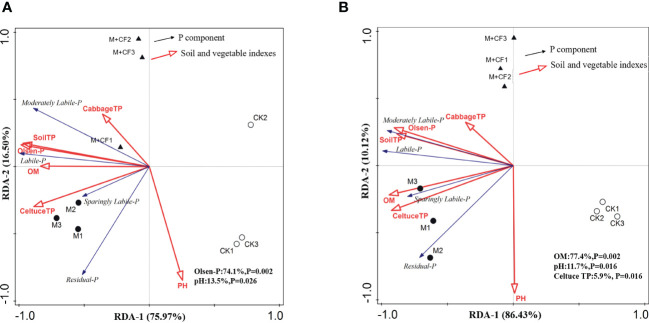
Relationship between factors of soil, vegetable and different P fractions response to different fertilizer applications at two soil layers. Labile-P: Resin-P, NaHCO_3_-Pi, NaHCO_3_-Po; Mod. Labile-P: NaOH-Pi, NaOH-Po, HCl-Pi; Spa.Labile-P: Conc.HCl-Pi, Conc.HCl-Po; Residual-P. **(A)** represents 0-20 cm soil layer, **(B)** represents 20-40 cm soil layer.

The three treatments were divided into manure application (M and M+CF) and control (CK) groups by the RDA (**Figure 4**). The manure application (M and M+CF) treatments were positively correlated with the soil-vegetable P variables and P fractions, whereas the control treatment (CK) was negatively correlated with these same variables, except for soil pH. Soil Olsen-P concentration and pH were the most important factors explaining the variation in the P fractions in response to manure application; specifically, they explained 87.6% of the variation at the 0–20-cm soil layer (**Figure 4A**). Olsen-P alone explained 74.1% (*P = 0.002*) of the variation, whereas soil pH explained 13.5% (*P = 0.03*). Moreover, soil OM concentration, pH, and lettuce TP concentration were the three key factors that affected the variation in the P fractions in response to manure application at the 20–40-cm soil layer. Finally, soil OM concentration explained 77.4% (*P = 0.002*), pH explained 11.7% (*P = 0.02*), and lettuce TP explained 5.9% (*P = 0.02*) of the total variation (**Figure 4B**).

### 3.7 Relationships of available P and P fractions with manure application

**Figure 5:**
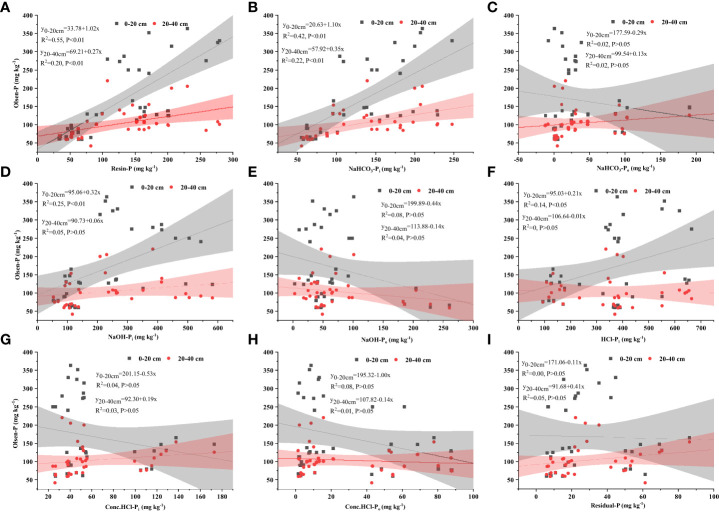
Relationships between available P (Olsen-P) concentration and soil labile-P: Resin-P **(A)**, NaHCO_3_-P_i_ **(B)**, NaHCO_3_-P_o_ **(C)**; Moderately Labile-P: NaOH-P_i_ **(D)**, NaOH-P_o_ **(E)**, HCl-P_i_ **(F)** and Sparingly Labile-P: Conc.HCl-P_i_ **(G)**, Conc.HCl-P_o_ **(H)** and Residual-P **(I)**, at 0-20 cm, 20-40 cm soil depth, per layer for all 36 samples of 3 treatments in the current study. *P* indicates significant regressions at< *0.01*,*< 0.05* or *> 0.05* levels, respectively.

The soil-available P (Olsen-P) concentration increased with increasing soil labile-P. That is, it increased as resin-P (*R* ^2^ = 0.55, *P< 0.01*, **Figure 5A**), NaHCO_3_-P_i_ concentration (*R* ^2^ = 0.42, *P< 0.01*, **Figure 5B**), and moderately labile-P increased, as well as NaOH-P_i_ (*R* ^2^ = 0.25, *P< 0.01*, **Figure 5D**) and HCl-P_i_ (*R* ^2^ = 0.14, *P< 0.05*, **Figure 5F**) concentration at the 0–20-cm soil layer. In contrast, the soil-available P (Olsen-P) concentration only increased with an increase in soil labile-P, i.e., resin-P (*R* ^2^ = 0.20, *P< 0.01*) and NaHCO_3_-P_i_ concentration (*R* ^2^ = 0.22, *P< 0.01*) at the 20–40-cm soil layer (**Figures 5A, B**). Changes of other P fractions i.e., NaHCO3-Po, NaOH-Po, Conc.HCl-Pi, Conc.HCl-Po, Residual-P concentration (**Figures 5C, E, G–I**) did not significantly affect soil-available P (Olsen-P) concentration

### 3.8 Vegetable yield and vegetable P concentration with manure application

**Figure 6:**
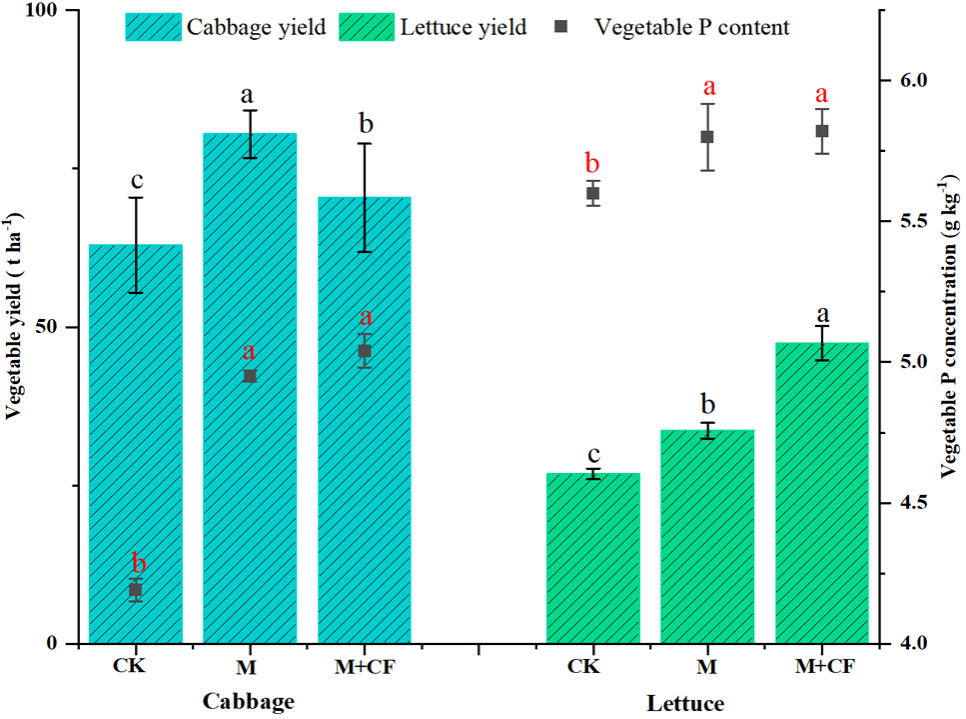
Vegetable yield and vegetable P concentration. Bars over the marker show standard error (n=3). Black different letters above the columns indicate significant differences in vegetable yield at *P*<0.05, and red different letters above the squares indicate significant differences in vegetable P concentration at *P*<0.05.

Given the P input, the cabbage yield was 1.5–2.4 times higher than the lettuce yield (*P< 0.05*, **Figure 6**). Furthermore, the cabbage yield under the M treatment (80.45 ± 3.76 t ha^-1^) was higher than that under M+CF (70.42 ± 8.53 t ha^-1^, *P< 0.05*), and its PUE under the M treatment (47.79%) was also higher compared to that under M+CF (42.62%) (*P< 0.05*, **Table 2**). However, the lettuce yield under the M+CF treatment (47.44 ± 2.72 t ha^-1^) was higher compared to that under M (33.64 ± 1.25 t ha^-1^, *P< 0.05*), and its PUE (33.13%) under M+CF was higher compared to that under M (23.41%, *P< 0.05*). Cabbage (62.94 ± 7.52 t ha^-1^) and lettuce yields (26.80 ± 0.86 t ha^-1^) were lowest under the control treatment (*P< 0.05*). The cabbage P concentration ranged from 4.19–5.04 g kg^-1^, whereas the lettuce P concentration ranged from 5.6–5.82 g kg^-1^. Finally, the lettuce P concentration was higher compared to that of cabbage (*P< 0.05*).

### 3.9 P surpluses and environmental risk of P loss

#### 3.9.1 Relationship between available P and P surpluses with manure application

**Figure 7:**
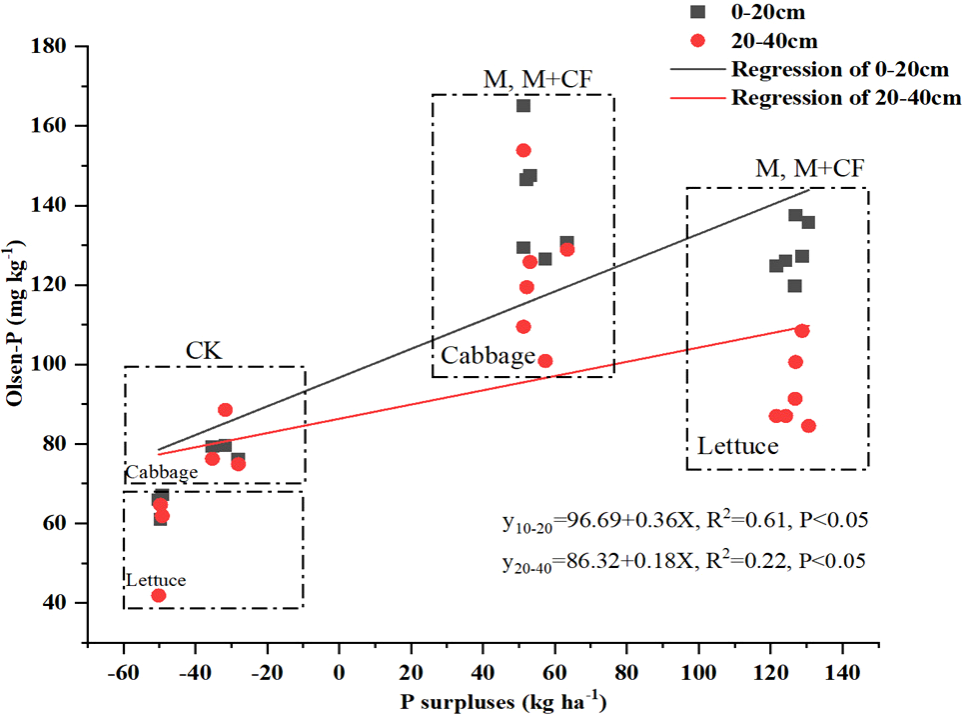
Relationships between seasonal P surpluses and available-P (Olsen-P) concentration at two soil layers, per layer for 18 samples of 3 treatments after harvest seasons of the current study. P indicates significant regressions at < 0.05 level..

A strong positive correlation was observed between seasonal P surpluses (i.e., P_surplus_ = P_input_ − P_output_) and soil available-P (Olsen-P) enrichment after the harvest seasons in both soil layers (y_10-20_ = 96.69 + 0.36X, *R* ^2^ = 0.61, *P< 0.05*; y_20-40_ = 86.32 + 0.18X, *R* ^2^ = 0.22, *P< 0.05*) (**Figure 7**). Lettuce had higher P surpluses in two treatments (M and M+CF) compared to cabbage for the same annual P input amount, except for the CK treatment. With fertilizer application, more Olsen-P accumulated at the 0–20-cm soil layer in two of the treatments (M and M+CF), whereas the Olsen-P concentration was higher in cabbage compared to lettuce under the no-fertilizer application treatment (CK).

#### 3.9.2 Relationship between available P and CaCl_2_-P with manure application

**Figure 8:**
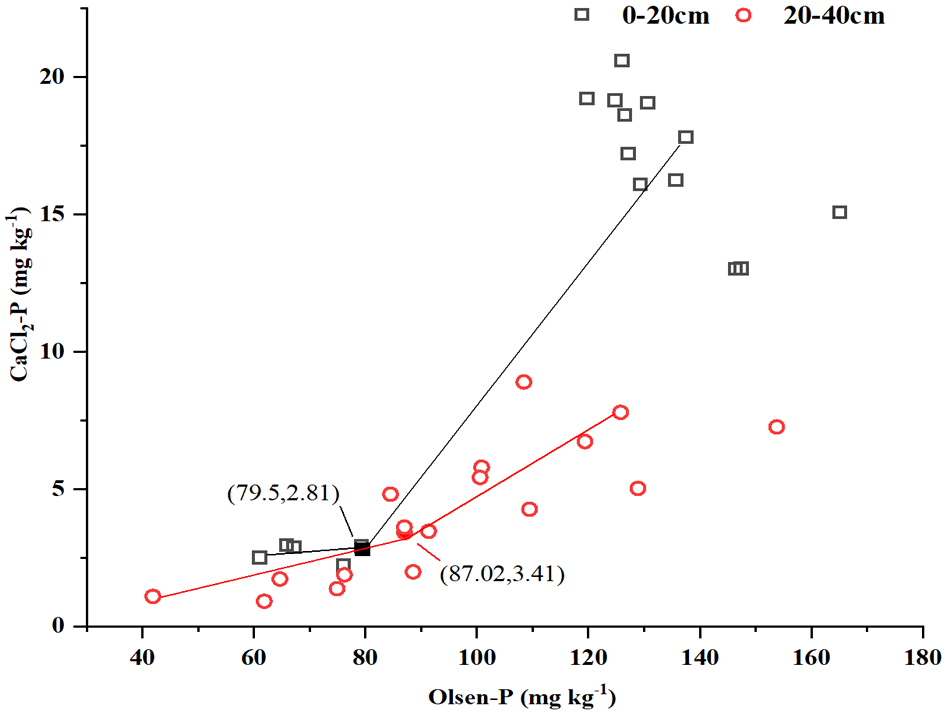
Relationships between CaCl_2_-P concentration and available-P (Olsen-P) at two soil layers for 3 treatments in the current study. The coordinates for the significant change points are indicated in parentheses; the first number indicates the X value, and the second number indicates the Y value.

An inflexion point (breakpoint) model was created to describe the relationship between CaCl_2_-P and available P (Olsen-P) (**Figure 8**). Above the inflexion point, a dramatic increase occurred in CaCl_2_-P as the Olsen-P concentration increased. The inflexion points of soil Olsen-P and CaCl_2_-P at the two soil layers were 79.5 mg kg^-1^ and 87.02 mg kg^-1^, respectively.

## 4 Discussion

### 4.1 Long-term manure application increases PUE and yield in a subtropical region

The global average PUE for fruits and vegetables is approximately 22% (2015–2019 average), which is much lower compared to those of common crops (above 50%) and the levels obtained in China (less than 22%) (Zou et al., 2022). By calculating P input, output, and PUE in a subtropical highland monsoon-type climate, we found that the annual PUE of the 14-year open-field vegetable system was about 36%–38%, which is higher compared to the global average. This is also higher compared to the 15%–16% of the 10–15-year open-field vegetable system (OM > 10 g kg^-1^, pH = 5.29) in Chongqing Province, southwest China, which is also characterized by a typical subtropical monsoon climate (Zhang et al., 2021). Acidic soils with a strong P absorption capacity (OM = 14.5–19.6 g kg^-1^, pH = 4.2–4.7), limited available P, and low PUE (13%–16%) have been reported for the southwestern Fujian Province, China, which is characterized by a southern subtropical monsoon climate (Chen et al., 2022). Thus, a higher soil OM concentration (42.37–59.92 g kg^-1^) and pH (7.1–7.4) in the 0–20-cm soil layer can explain the higher PUE observed in our study of the Erhai watershed in Yunnan Province, southwest China.

Previous studies have shown that long-term single manure application/or control (no fertilizer) increased the pH of the soil and buffered the process of soil acidification (Shi et al., 2019). However, the surplus P of long-term single manure application in soil increased and tended to move down the soil, posing a serious threat to environmental quality (Qin et al., 2020). In our study, M+CF caused soil acidification, mainly because after many years of crop planting and harvest (the whole-year yield of M+CF was higher than that obtained with a single manure application of M), more salt-based ions, such as Ca^2+^ and Mg^2+^, were removed, which were not supplemented, and the nutrients were imbalanced, resulting in the loss of salt-based ions and the residue of H^+^, with lower pH values (Guo et al., 2010). To maintain the stability of soil pH and the sustainability of the planting environment, soil testing formula fertilization can be used to enhance crop nutrient uptake and rationally allocate crop rotation, with the aim to reduce environmental pollution and develop a sustainable agriculture.

Furthermore, the vegetable yields of both M and M+CF treatments increased with manure application; however, manure application was more conducive to an increased leafy and fruity vegetable yield in contrast to stem vegetables. This is due to the former having shallow root systems and a low root density, which enables them to easily assimilate as much available P as possible at the topsoil level (Xiang et al., 2022). These results agree with the findings of our study, namely that cabbage was more sensitive to manure application than lettuce, whereas cabbage yield and PUE were both enhanced compared with lettuce (**Figure 6**, **Table 3**). However, this study was limited by data availability and scope and did not take the root exudates and microbial biomass P into account. At the sametime, this manuscript assumed P input (Fertilizer and manure) to be same annually throughout the study period and the nutrient concentrations for manure represent the average values over years. The limited sampling size also weakened the power of this PUE for interpretation of the effects of long-term manure application on soil biological properties. Thus, further studies should take these factors into account under long-term manure application.

**Table 3:** Mean annual P output and PUE of two vegetables among three treatments.

Treatment	Cabbage			Lettuce		
	P_input_	P_output_	PUE	P_input_	P_output_	PUE
	kg·ha^−1^	kg·ha^−1^	%	kg·ha^−1^	kg·ha^−1^	%
CK	0	31.67 ± 3.6b	/	0	18.01 ± 0.5c	/
M	100	47.79 ± 0.9a	47.79 ± 0.9a	100	23.41 ± 1.8b	23.41 ± 1.8b
M+CF	100	42.62 ± 6.1a	42.62 ± 6.1a	100	33.13 ± 2.6a	33.13 ± 2.6a

Data are means standard errors (n=14 years), the different letters in the same column mean significant differences at P<0.05, respectively. CK is a control treatment.

### 4.2 Effects of long-term manure application on the Pi pool

Manure application increased the P_i_ concentration, which ranged between 2% and 72% as well as 9% and 68% at the two soil depths (**Figure 3**). This result was similar to the findings of a previous study (Yan et al., 2016), in which manure application significantly increased soil P_i_ in the 0–30-cm layer in a 9-year tomato double-cropping system. Specifically, manure application largely increased the proportion of labile-P (resin-P and NaHCO_3_-P_i_), in contrast to moderately labile-P, by 32%–72% (**Figure 3**). This is in line with previous studies (Yan et al., 2016) demonstrating that manure application in intensive vegetable cropping systems characterized by calcareous soil significantly increased the soil orthophosphate concentration ( ${\text{H}}_{2}{\text{PO}}_{4}^{-}$ or ${\text{HPO}}_{4}^{2-}$ ), which can be assimilated directly by plants. Qin et al. (2020) further clarified the main reasons for the orthophosphate increase with manure application in the wheat-maize rotation system in the North China Plain. Specifically, the authors claimed this to be due to a considerable amount of orthophosphate in manure, as well as the conversion of P_o_ to P_i_ in the soil. This can be explained as follows: resin-P and NaHCO_3_-P_i_ are the dominant available P_i_ fractions in manure, whereas the moderately labile-P concentration depends on the rate of mineralization and transformation from P_o_ to P_i_ and can be accelerated by manure application (Qin et al., 2020). Organic amendments increase the available P by inhibiting Ca phosphate precipitation, decreasing the precipitation rate of poorly soluble phosphate, and mobilizing native soil P (Mohanty et al., 2006; Mao et al., 2015). Additionally, moderately labile-P (NaOH-P_i_) was the main P_i_ component extracted by NaOH (**Figure 1**) and also the secondary mineral P_i_ that was extracted, including potentially labile Al-P/Fe-P, whereas the only P_i_ component extracted by diluted HCl (HCl-P_i_) was potentially labile mineral P_i_ (i.e., Ca-P; Zhang et al., 2020). Moreover, the soil P input obtained *via* chemical fertilizers was easily remedied by Al^3+^ and Fe^2+^ in acidic soils or absorbed by Ca^2+^ and Mg^2+^ in alkaline soils (Penn and Camberato, 2019; Naeem et al., 2021).

Furthermore, an increased soil pH reduced the adsorption capacity of soil P and increased soil P availability (Abdala et al., 2012). In this study, the pH of the M+CF treatment (4.3/4.3) at the two soil depths was lower compared to the those in the M (7.1/7.2) and control (7.4/6.9) treatments (**Table 1**). Thus, the M+CF treatment had a higher NaOH-P_i_ (Fe-P/Al-P) and lower HCl-P_i_ (Ca-P/Mg-P) concentration (**Figure 3**). Additionally, OM addition induced soil microbial P cycling (Lin et al., 2019), and it is therefore likely that an increased OM concentration that results from manure application facilitates the transformation of moderately labile-P to labile-P, which eventually caused an increased accumulation of available P (Olsen-P) at the 0–20-cm soil layer (**Table 1**). Finally, the proportion of moderately labile-P (HCl-P_i_) decreased with manure application (**Figure 3**).

### 4.3 Effects of long-term manure application on the P_o_ pool

The increased P_o_ concentration ranged between 5–61% and 8–61% for the two soil layers; these ranges were lower than those compared to P_i_ (**Figure 4**). Overall, long-term manure application increased the pools of P_o_, labile-P (e.g., NaHCO_3_-P_o_), moderately labile-P (e.g., NaOH-P_o_), and low-labile-P (e.g., Conc.HCl-P_o_) to a greater extent at the 20–40-cm soil layer compared to the 0–20-cm layer, except for NaOH-P_o_ from the M+CF treatment at the 0–20-cm layer (**Figure 4**).

It is possible that a lower OM concentration decreased the NaOH-P_o_ transformation rate, thereby resulting in a higher NaOH-P_o_ proportion. A previous study indicated that topsoil contained a high OM concentration and thus had a higher biological abundance and activity (Song et al., 2017). A higher OM enhances microbial activity, induces nutrient cycling driven by soil microbes (Lin et al., 2019), and increases the activity of soil phosphomonoesterase (PME), indicating that mineralization rates have changed from P_o_ to available P_i_ (Nakayama et al., 2021). Additionally, the release of organic acids into the surrounding soil, as well as other microbial secondary metabolites, due to long-term manure application also affected the soil P fractions and their availability (Malik et al., 2012). Furthermore, OM replaced P sorption sites and reduced P retention, which resulted in an improved P availability (Bhatti et al., 1998). In the absence of a long-term fertilizer supply, the P_o_ pool started to be mineralized and thereby provided available P (Randhawa et al., 2005). Aulakh et al. (2003) also reported that the P_o_ and P_i_ pools acted as a continuous quasi-equilibrium of the total soil P pool, which can be regulated by the activities of above- and belowground organisms. Thus, long-term manure application facilitates the transformation of NaOH-P_o_ to P_i_ and results in a lower NaOH-P_o_ concentration at the 0–20-cm soil layer under manure treatment (M and M+CF, **Figure 4**). This may also be related to the low OM concentration in the control treatment (**Table 1**).

In contrast, the proportion of moderately labile-P (NaOH-P_o_), as opposed to labile-P (NaHCO_3_-P_o_) and low-labile-P (Conc.HCl-P_o_), increased at the 20–40-cm soil layer with long-term single manure application (**Figure 3**). This indicates that, in the subsoils of a subtropical open-field vegetable system, moderately labile P_o_ is more likely to accumulate with manure application compared to labile- and low-labile- P_o_. This is likely related to a decreased OM concentration in the subsoil (Song et al., 2017) or due to the relatively long conversion period of P from manure within the subsoil (Halajnia et al., 2009). This result is in line with a previous study which demonstrated that P provided by manure amendment was a sustainable slow-release P source for crops (Mao et al., 2015).

### 4.4 Effects of long-term manure application on the available-P (Olsen-P) pool

In the major vegetable production regions of China, the mean soil Olsen-P concentrations in the 0–20-cm soil layers of greenhouse and open-field vegetable production systems are 179 and 100 mg P kg^-1^, respectively. In general, the threshold soil Olsen-P level for vegetable production is 60 mg P kg^-1^. In contrast, this value is 34 mg P kg^-1^ in cereal production systems (Yan et al., 2013). The Olsen-P concentrations with manure application (M and M+F) in our open-field vegetable system are higher than the mean Olsen-P value (100 mg P kg^-1^) in China, and CK (64.69 mg P kg^-1^) was above the critical value (60 mg P kg^-1^) at both soil layers. With an increase in seasonal P surpluses (i.e., input–crop removal), soil Olsen-P was greatly enriched (Yan et al., 2013), and soil CaCl_2_-P therefore served as an indicator that the potential P leaching loss increased dramatically with Olsen-P (Liang et al., 2009; Wang et al., 2012).

We also observed a positive relationship between seasonal P surpluses and soil Olsen-P (**Figure 7**), which is similar to the results of Yan et al. (2013). These authors also found such a positive relationship in several intensive vegetable fields in Beijing, Shouguang, and Shijiazhuang. The topsoil Olsen-P concentration was higher compared to that of the subsoil with manure application (M and M+F), whereas the Olsen-P concentration under the M treatment was higher compared to that under the M+F treatment (**Table 1**). This implies that manure application alone might potentially increase the risk of P loss (Qin et al., 2020). Compared with cabbage, lettuce production resulted in more P surpluses at the 0–20-cm and 0–40-cm soil layers (**Figure 7**). This might explain why cabbage had a higher PUE than lettuce (**Figure 6**, **Table 3**). This has important implications for sustainable lettuce production, suggesting that less P fertilizer should be applied, particularly if it is to be obtained from manure. The inflexion point (breakpoint) differed between soil Olsen-P and CaCl_2_-P at the two soil layers (**Figure 8**) (Wang et al., 2012). Specifically, the topsoil Olsen-P concentration was 79.5 mg kg^-1^ and thus had a higher loss risk compared to the subsoil (87.02 mg kg^-1^) (**Figure 8**).

## 5 Conclusion

This 14-year long-term manure experiment in a subtropical highland vegetable system revealed that M application can increase the concentration of P fractions at the 0–40-cm soil, except for moderately labile-P (NaOH-Pi). Furthermore, M+F can help to reduce the amount of residual-P and may represent an optimized long-term yield approach. However, this approach should be combined with corresponding management measures, such as soil testing formula fertilization and rational crop rotation allocation, to avoid soil acidification and ensure sustainability in subtropical vegetable systems. Our findings suggest that careful attention should be given to the P balance to avoid excessive P input, especially in the case of stem vegetables with manure application. Implementing a rational manure application strategy can decrease the environmental risks posed by P loss in vegetable systems in China.

## Data availability statement

The original contributions presented in the study are included in the article/supplementary material. Further inquiries can be directed to the corresponding author.

## Author contributions

YM: Conceptualization, methodology, investigation, writing–original draft, formal analysis, visualization. WH: Methodology, data curation, visualization. YML: Conceptualization, methodology, writing–review. YL: Writing–review & editing, validation, visualization. BL: Conceptualization, writing–review & editing, supervision, project administration, funding acquisition. YZ: Conceptualization, resources, writing–review & editing, supervision, validation. All authors contributed to the article and approved the submitted version.
